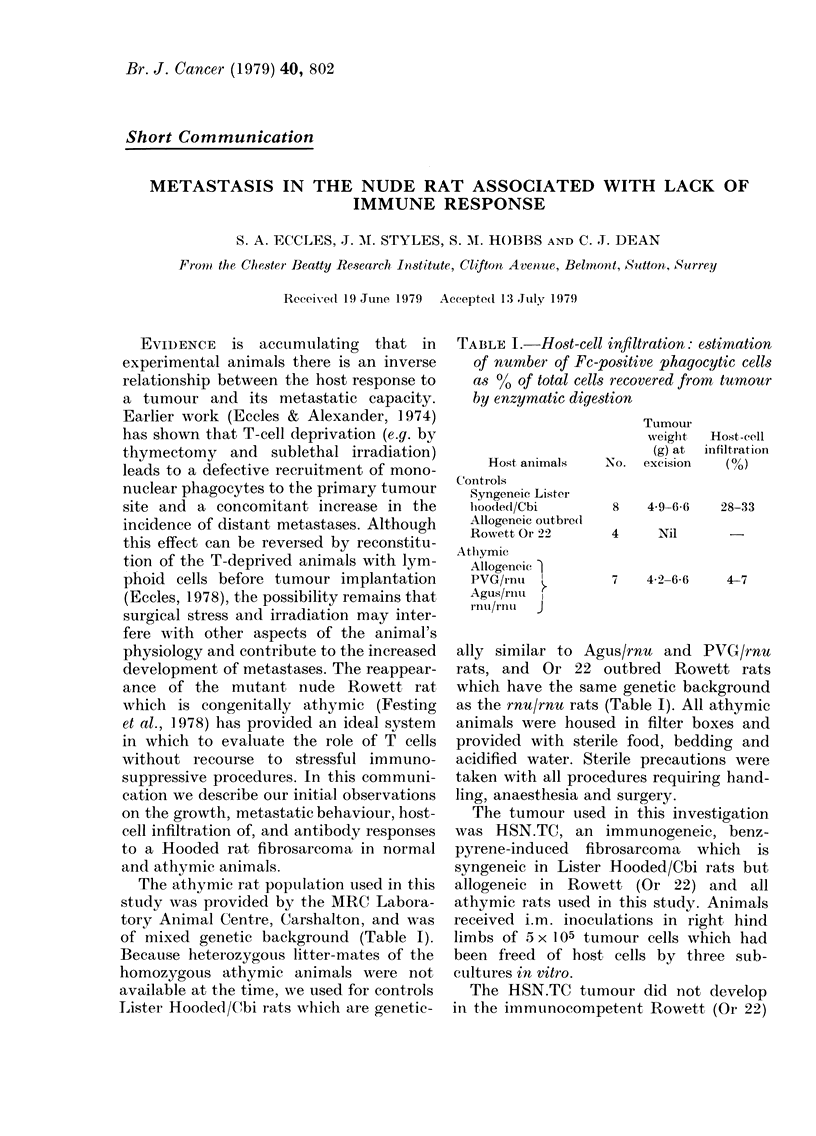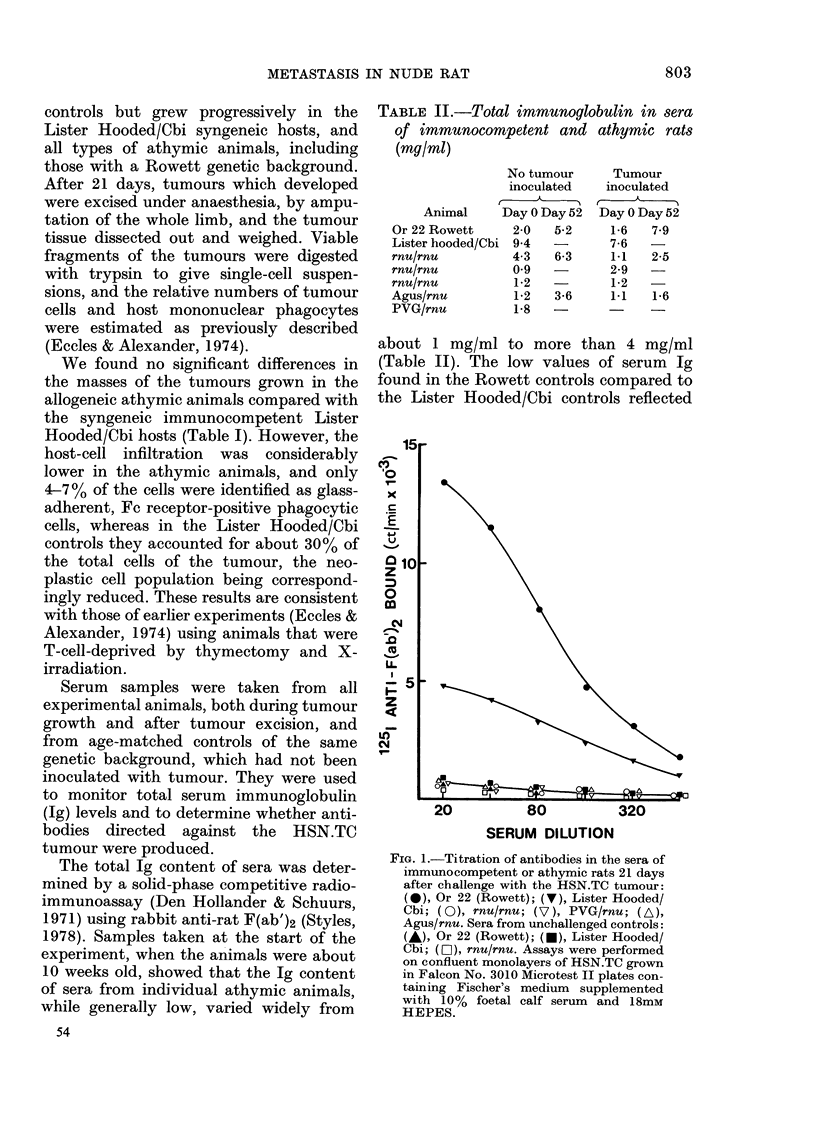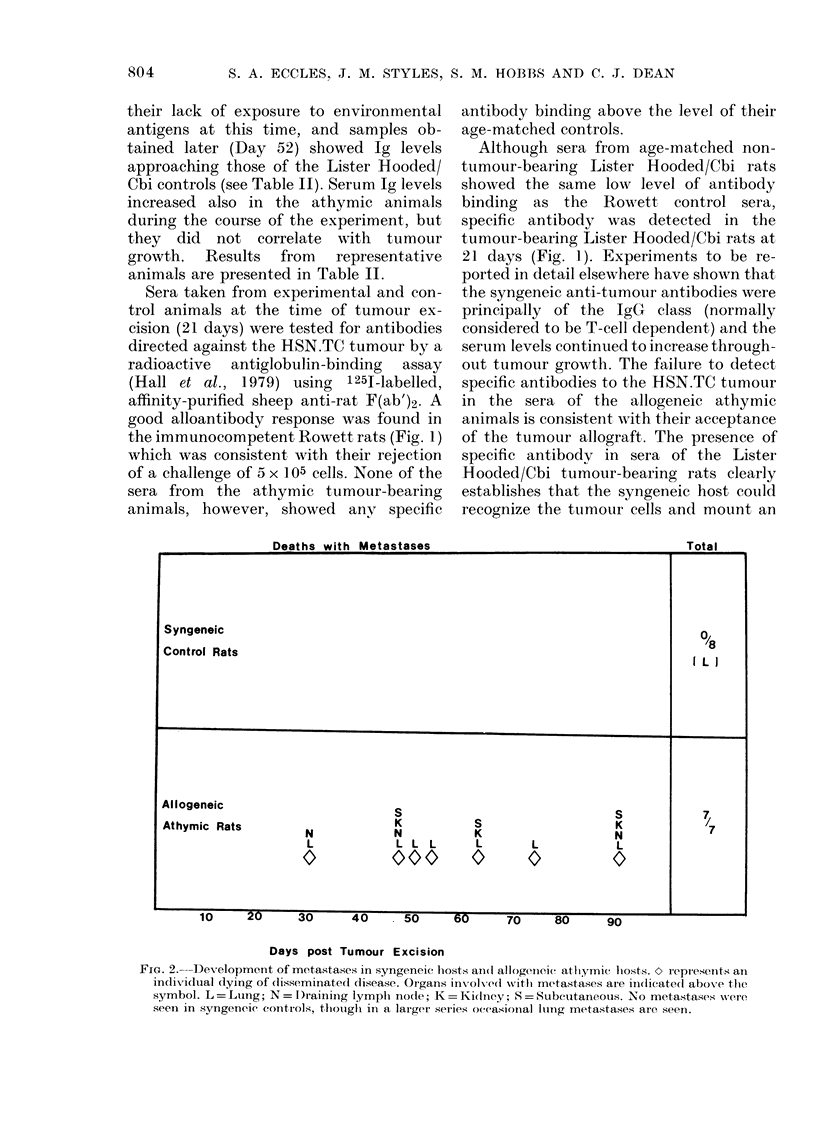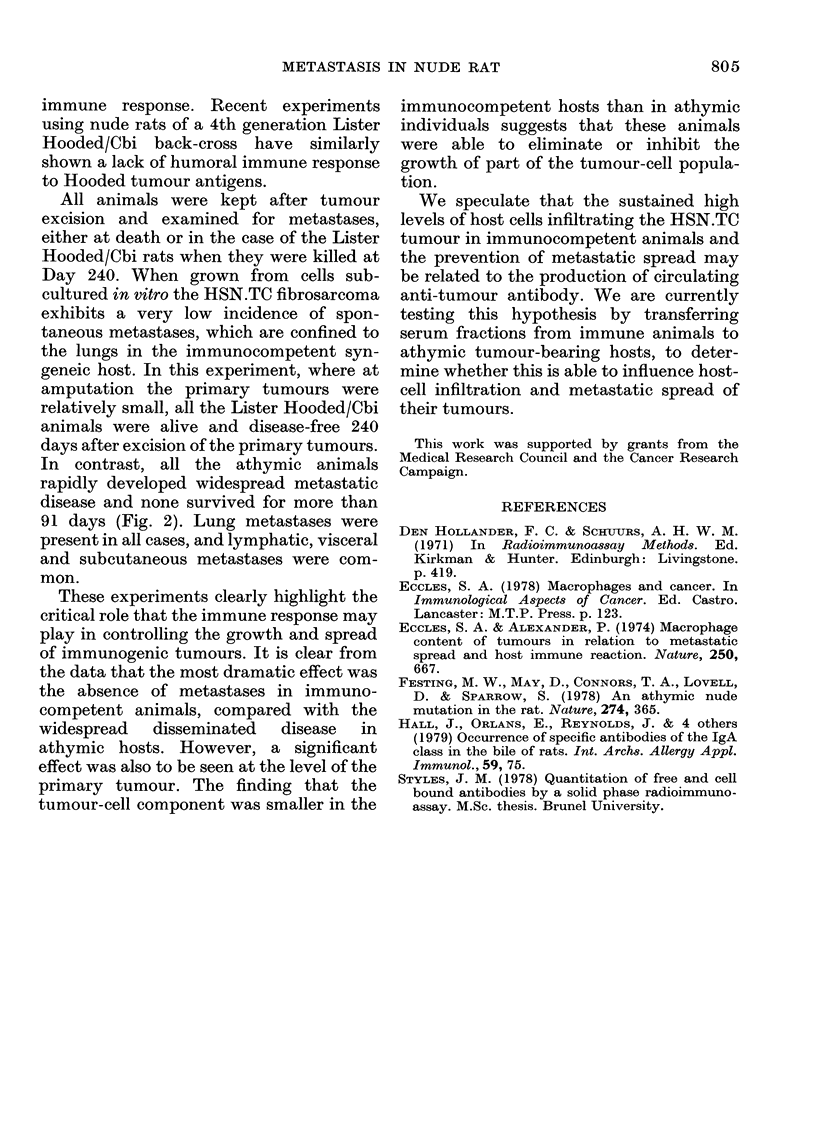# Metastasis in the nude rat associated with lack of immune response.

**DOI:** 10.1038/bjc.1979.264

**Published:** 1979-11

**Authors:** S. A. Eccles, J. M. Styles, S. M. Hobbs, C. J. Dean


*Br. J. Cancer* (1979) **40,** 802

***Short Communication***

### METASTASIS IN THE NUDE RAT ASSOCIATED WITH LACK OF IMMUNE RESPONSE

S. A. ECCLES, J. M. STYLES, S. M. HOBBS AND C. J. DEAN

*From the Chester Beatty Research Institute, Clifton Avenue, Belmont, Sutton, Surrey*

Received 19 June 1979 Accepted 13 July 1979

EVIDENCE is accumulating that in experimental animals there is an inverse relationship between the host response to a tumour and its metastatic capacity. Earlier work (Eccles & Alexander, 1974) has shown that T-cell deprivation (*e.g.* by thymectomy and sublethal irradiation) leads to a defective recruitment of mononuclear phagocytes to the primary tumour site and a concomitant increase in the incidence of distant metastases. Although this effect can be reversed by reconstitution of the T-deprived animals with lymphoid cells before tumour implantation (Eccles, 1978), the possibility remains that surgical stress and irradiation may interfere with other aspects of the animal's physiology and contribute to the increased development of metastases. The reappearance of the mutant nude Rowett rat which is congenitally athymic (Festing *et al.*, 1978) has provided an ideal system in which to evaluate the role of T cells without recourse to stressful immunosuppressive procedures. In this communication we describe our initial observations on the growth, metastatic behaviour, host-cell infiltration of, and antibody responses to a Hooded rat fibrosarcoma in normal and athymic animals.

The athymic rat population used in this study was provided by the MRC Laboratory Animal Centre, Carshalton, and was of mixed genetic background (Table I). Because heterozygous litter-mates of the homozygous athymic animals were not available at the time, we used for controls Lister Hooded/Cbi rats which are genetically similar to Agus/*rnu* and PVG/*rnu* rats, and Or 22 outbred Rowett rats which have the same genetic background as the *rnu*/*rnu* rats (Table I). All athymic animals were housed in filter boxes and provided with sterile food, bedding and acidified water. Sterile precautions were taken with all procedures requiring handling, anaesthesia and surgery.

TABLE I.—*Host-cell infiltration: estimation of number of Fc-positive phagocytic cells as % of total cells recovered from tumour by enzymatic digestion*

| Host animals | No. | Tumour weight (g) at excision | Host-cell infiltration (%) |
|---|---|---|---|
| Controls | | | |
| Syngeneic Lister hooded/Cbi | 8 | 4·9–6·6 | 28–33 |
| Allogeneic outbred Rowett Or 22 | 4 | Nil | — |
| Athymic | | | |
| Allogeneic PVG/rnu, Agus/rnu, rnu/rnu | 7 | 4·2–6·6 | 4–7 |

The tumour used in this investigation was HSN.TC, an immunogeneic, benzpyrene-induced fibrosarcoma which is syngeneic in Lister Hooded/Cbi rats but allogeneic in Rowett (Or 22) and all athymic rats used in this study. Animals received i.m. inoculations in right hind limbs of $5 \times 10^5$ tumour cells which had been freed of host cells by three subcultures *in vitro*.

The HSN.TC tumour did not develop in the immunocompetent Rowett (Or 22)

controls but grew progressively in the Lister Hooded/Cbi syngeneic hosts, and all types of athymic animals, including those with a Rowett genetic background. After 21 days, tumours which developed were excised under anaesthesia, by amputation of the whole limb, and the tumour tissue dissected out and weighed. Viable fragments of the tumours were digested with trypsin to give single-cell suspensions, and the relative numbers of tumour cells and host mononuclear phagocytes were estimated as previously described (Eccles & Alexander, 1974).

We found no significant differences in the masses of the tumours grown in the allogeneic athymic animals compared with the syngeneic immunocompetent Lister Hooded/Cbi hosts (Table I). However, the host-cell infiltration was considerably lower in the athymic animals, and only 4–7% of the cells were identified as glass-adherent, Fc receptor-positive phagocytic cells, whereas in the Lister Hooded/Cbi controls they accounted for about 30% of the total cells of the tumour, the neoplastic cell population being correspondingly reduced. These results are consistent with those of earlier experiments (Eccles & Alexander, 1974) using animals that were T-cell-deprived by thymectomy and X-irradiation.

Serum samples were taken from all experimental animals, both during tumour growth and after tumour excision, and from age-matched controls of the same genetic background, which had not been inoculated with tumour. They were used to monitor total serum immunoglobulin (Ig) levels and to determine whether antibodies directed against the HSN.TC tumour were produced.

The total Ig content of sera was determined by a solid-phase competitive radioimmunoassay (Den Hollander & Schuurs, 1971) using rabbit anti-rat F(ab′)2 (Styles, 1978). Samples taken at the start of the experiment, when the animals were about 10 weeks old, showed that the Ig content of sera from individual athymic animals, while generally low, varied widely from

TABLE II.—*Total immunoglobulin in sera of immunocompetent and athymic rats (mg/ml)*

| Animal | No tumour inoculated | | Tumour inoculated | |
|---|---|---|---|---|
| | Day 0 | Day 52 | Day 0 | Day 52 |
| Or 22 Rowett | 2·0 | 5·2 | 1·6 | 7·9 |
| Lister hooded/Cbi | 9·4 | — | 7·6 | — |
| *rnu/rnu* | 4·3 | 6·3 | 1·1 | 2·5 |
| *rnu/rnu* | 0·9 | — | 2·9 | — |
| *rnu/rnu* | 1·2 | — | 1·2 | — |
| Agus/*rnu* | 1·2 | 3·6 | 1·1 | 1·6 |
| PVG/*rnu* | 1·8 | — | — | — |

about 1 mg/ml to more than 4 mg/ml (Table II). The low values of serum Ig found in the Rowett controls compared to the Lister Hooded/Cbi controls reflected

FIG. 1.—Titration of antibodies in the sera of immunocompetent or athymic rats 21 days after challenge with the HSN.TC tumour: (●), Or 22 (Rowett); (▼), Lister Hooded/Cbi; (○), *rnu/rnu*; (▽), PVG/*rnu*; (△), Agus/*rnu*. Sera from unchallenged controls: (▲), Or 22 (Rowett); (■), Lister Hooded/Cbi; (□), *rnu/rnu*. Assays were performed on confluent monolayers of HSN.TC grown in Falcon No. 3010 Microtest II plates containing Fischer's medium supplemented with 10% foetal calf serum and 18mM HEPES.

their lack of exposure to environmental antigens at this time, and samples obtained later (Day 52) showed Ig levels approaching those of the Lister Hooded/Cbi controls (see Table II). Serum Ig levels increased also in the athymic animals during the course of the experiment, but they did not correlate with tumour growth. Results from representative animals are presented in Table II.

Sera taken from experimental and control animals at the time of tumour excision (21 days) were tested for antibodies directed against the HSN.TC tumour by a radioactive antiglobulin-binding assay (Hall *et al.*, 1979) using $^{125}$I-labelled, affinity-purified sheep anti-rat $F(ab')_2$. A good alloantibody response was found in the immunocompetent Rowett rats (Fig. 1) which was consistent with their rejection of a challenge of $5 \times 10^5$ cells. None of the sera from the athymic tumour-bearing animals, however, showed any specific antibody binding above the level of their age-matched controls.

Although sera from age-matched non-tumour-bearing Lister Hooded/Cbi rats showed the same low level of antibody binding as the Rowett control sera, specific antibody was detected in the tumour-bearing Lister Hooded/Cbi rats at 21 days (Fig. 1). Experiments to be reported in detail elsewhere have shown that the syngeneic anti-tumour antibodies were principally of the IgG class (normally considered to be T-cell dependent) and the serum levels continued to increase throughout tumour growth. The failure to detect specific antibodies to the HSN.TC tumour in the sera of the allogeneic athymic animals is consistent with their acceptance of the tumour allograft. The presence of specific antibody in sera of the Lister Hooded/Cbi tumour-bearing rats clearly establishes that the syngeneic host could recognize the tumour cells and mount an

FIG. 2.—Development of metastases in syngeneic hosts and allogeneic athymic hosts. ◇ represents an individual dying of disseminated disease. Organs involved with metastases are indicated above the symbol. L = Lung; N = Draining lymph node; K = Kidney; S = Subcutaneous. No metastases were seen in syngeneic controls, though in a larger series occasional lung metastases are seen.

immune response. Recent experiments using nude rats of a 4th generation Lister Hooded/Cbi back-cross have similarly shown a lack of humoral immune response to Hooded tumour antigens.

All animals were kept after tumour excision and examined for metastases, either at death or in the case of the Lister Hooded/Cbi rats when they were killed at Day 240. When grown from cells subcultured *in vitro* the HSN.TC fibrosarcoma exhibits a very low incidence of spontaneous metastases, which are confined to the lungs in the immunocompetent syngeneic host. In this experiment, where at amputation the primary tumours were relatively small, all the Lister Hooded/Cbi animals were alive and disease-free 240 days after excision of the primary tumours. In contrast, all the athymic animals rapidly developed widespread metastatic disease and none survived for more than 91 days (Fig. 2). Lung metastases were present in all cases, and lymphatic, visceral and subcutaneous metastases were common.

These experiments clearly highlight the critical role that the immune response may play in controlling the growth and spread of immunogenic tumours. It is clear from the data that the most dramatic effect was the absence of metastases in immunocompetent animals, compared with the widespread disseminated disease in athymic hosts. However, a significant effect was also to be seen at the level of the primary tumour. The finding that the tumour-cell component was smaller in the immunocompetent hosts than in athymic individuals suggests that these animals were able to eliminate or inhibit the growth of part of the tumour-cell population.

We speculate that the sustained high levels of host cells infiltrating the HSN.TC tumour in immunocompetent animals and the prevention of metastatic spread may be related to the production of circulating anti-tumour antibody. We are currently testing this hypothesis by transferring serum fractions from immune animals to athymic tumour-bearing hosts, to determine whether this is able to influence host-cell infiltration and metastatic spread of their tumours.

This work was supported by grants from the Medical Research Council and the Cancer Research Campaign.

### REFERENCES

Den Hollander, F. C. & Schuurs, A. H. W. M. (1971) In *Radioimmunoassay Methods*. Ed. Kirkman & Hunter. Edinburgh: Livingstone. p. 419.

Eccles, S. A. (1978) Macrophages and cancer. In *Immunological Aspects of Cancer*. Ed. Castro. Lancaster: M.T.P. Press. p. 123.

Eccles, S. A. & Alexander, P. (1974) Macrophage content of tumours in relation to metastatic spread and host immune reaction. *Nature*, **250**, 667.

Festing, M. W., May, D., Connors, T. A., Lovell, D. & Sparrow, S. (1978) An athymic nude mutation in the rat. *Nature*, **274**, 365.

Hall, J., Orlans, E., Reynolds, J. & 4 others (1979) Occurrence of specific antibodies of the IgA class in the bile of rats. *Int. Archs. Allergy Appl. Immunol.*, **59**, 75.

Styles, J. M. (1978) Quantitation of free and cell bound antibodies by a solid phase radioimmunoassay. M.Sc. thesis. Brunel University.